# Mechanistic insights into the anti-aging effects of *Crocus sativus* in a D-Gal-induced *in vitro* neural senescence model

**DOI:** 10.1371/journal.pone.0320572

**Published:** 2025-07-16

**Authors:** Vanessa Castelli, Patrizia Cesare, Erjola Bej, Michele d’Angelo, Anna Rita Volpe

**Affiliations:** Department of Life, Health and Environmental Sciences, University of L’Aquila, L’Aquila, Italy; Shahrekord University of Medical Science, IRAN, ISLAMIC REPUBLIC OF

## Abstract

**Background:**

The increasing number of elderly individuals has made age-related disorders a significant health concern. Aging is a natural, progressive and gradual phenomenon that leads to irreversible modifications in all molecules, cells, tissues and organs of an organism. Brain senescence is associated with increased risk of developing various neurological diseases, such as Alzheimer’s disease, Parkinson’s disease, and stroke. Therefore, finding effective strategies to counteract or delay brain senescence is of great importance for improving the quality of life and health span of the elderly population. Previous studies demonstrated that D-galactose is an appropriate agent to induce aging effects in *in vivo* and *in vitro* models.

**Purpose:**

In the present study, we evaluated anti-aging effects of a local Saffron extract (SE from Central Italy) on D-GAL-induced aging model *in vitro*. Based on promising preliminary results, future studies will focus on testing this specific *Crocus sativus* stigma preparation in animal models of aging.

**Methods:**

The potential anti-aging effect was evaluated using assessment of cell proliferation, live-cell cytotoxicity, Beta-Galactosidase (β-GAL), lipid peroxidation, intracellular reactive oxygen species (ROS), advanced glycation end products (AGEs) and malondialdehyde (MDA) levels. Additionally, the effects of SE pretreatment were examined on cell cycle and endoplasmic reticulum stress. Additionally, we employed a novel approach to analyze deeper changes upon saffron extract treatment, which is label-free holotomography.

**Results:**

Overall, our findings suggested that pretreatment with SE was protective against D-GAL-induced senescence, by counteracting oxidative and endoplasmic reticulum stress and proteins that regulate cell death.

**Conclusion:**

We obtained interesting results since pre-treatment with SE ameliorated overall condition, and for the first time we observed the strong anti-aging effect of SE not only in term of morphology, but also dynamics and total dry mass of cells. Overall, our work introduces a novel and promising approach to counteract or delay brain senescence, potentially improving the quality of life and health span of the elderly population.

## 1. Introduction

Aging, being a natural progressive process responsible for irreversible changes to the cells, molecules, tissues and organs of every organism and debilitating losses of cellular function, can lead to the cascade of the pathogenesis of age-related disorders, as well as neurodegenerative disorders, such as Parkinson’s and Alzheimer’s disease [1].

Cellular senescence is a biological aging hallmark that exerts a crucial role in the development of neurodegenerative disorders [2,3]. It is characterized by alterations in morphology, gene expressions (transcriptional and epigenetic changes), and metabolic activities [1–3]. Brain senescence is the gradual decline of brain function and structure that occurs with aging. It is characterized by cognitive impairment, neurodegeneration, neuroinflammation, oxidative stress, and epigenetic changes [3]. This age-related redox imbalance is probably due to a weakened anti-oxidative defense system and the increased production of the reactive oxygen species such as superoxide, hydrogen peroxide and reactive nitric oxide [1]. Brain senescence is associated with increased risk of developing various neurological diseases, such as Alzheimer’s disease, Parkinson’s disease, and stroke [3]. Therefore, finding effective strategies to prevent or delay brain senescence is of great importance for improving the quality of life and health span of the elderly population.

Human SH-SY5Y cells are a well-employed cellular model for investigating the mechanistic aspects of neural development and neurodegeneration. D-galactose (D-Gal) is a reducing sugar that has been used to induce accelerated aging in *in vitro* and *in vivo* models. These studies suggest that D-galactose can induce senescence in different categories of brain cells by causing oxidative stress, DNA damage, inflammation, and apoptosis [4–6]. In this context, the use of antioxidants or natural compounds such as saffron could be relevant.

Saffron (*Crocus sativus L.*) is a spice derived from the flower of a perennial plant belonging to the Iridaceae family [7]. It has been utilized for centuries as a flavoring and coloring compound, as well as in traditional medicine [8]. Saffron contains numerous bioactive compounds, such as kaempferol, crocin, crocetin, safranal and picrocrocin, that showed potential benefits for human health in particular cognitive functions [9]. Recently, saffron and its constituents have attracted attention for their neuroprotective effects against brain senescence and related disorders [10]. Several clinical and preclinical studies have demonstrated that saffron and its constituents can improve cognitive functions, mood, sleep quality, and other neurological outcomes in patients with mild cognitive impairment and Alzheimer’s disease. Furthermore, saffron and its bioactive compounds showed neuroprotective effects through several mechanisms, including the modulation of neurotransmitters, enhanced neurogenesis, dampened neuroinflammation, lower oxidative stress, and so on [11]. Among the different bioactive molecules of saffron, crocin is the most abundant and characteristic, accountable for its bright red color and antioxidant properties. Crocin is known for its antioxidant, anti-inflammatory, and neuroprotective properties. It helps improve memory and mood but can cause gastrointestinal distress in high doses. Crocin is transformed to crocetin by hydrolysis in the gastrointestinal tract; crocetin, after reaching the bloodstream, is distributed in the tissues and thanks to the passive transcellular diffusion, can cross the blood brain barrier and reach the brain, which explains the central effects and the possible use in neurodegenerative diseases [12]. Meanwhile, the bioactivity of safranal is less known but since it can eliminate the free radicals and reduce the oxidative stress in the neurons, it may exert a positive effect on brain aging [7]. Moreover, safranal showed anticonvulsant, and antidepressant effects, however, high doses can lead to dizziness and headache.

Based on the reported evidence, the aim of our study was to evaluate the potential anti-aging effects of a local saffron extract on D-GAL-induced neural aging model using human SH-SY5Y cells.

Interestingly, for the first time, we observed a strong anti-aging effect of SE, not only in terms of antioxidant activity, but also improved cellular morphology, dynamics and total dry mass of the cells.

## 2. Materials and methods

### 2.1. Hydroalcoholic saffron extract preparation

Saffron (*C. sativus L.*) stigmas were collected in L’Aquila (Abruzzo, central Italy) in November 2022, and processed based on the tradition. Specifically, agriculture soil was prepared and then left to rest from November to August of the following year. The bulb, which germinated the previous year, reproduces in spring, generating two new bulbs. In August, the “best” bulbs are planted close together in one or two rows. Harvesting was carried out by hand during the flowering period - from mid-October to the first fortnight of November - every morning at dawn, to avoid the flowers opening with the sun. Saffron stigmas, which have a very distinctive sharp fragrance, were dried over oak wood embers on the same day they are harvested. The stigmas were then stored in small sterilized airtight pots and kept away from light at room temperature (RT). A *C. sativus* sample has been registered as CSVA-001 at the Herbarium Center of the Pharmacology and Neuropharmacology Lab at the University of L’Aquila, Italy.

To prepare the hydroalcoholic extract saffron dried stigmas (*C. sativus L.*) were weighed, ground and soaked in 70% EtOH for 2h at RT avoiding light and continuous stirring (250 mg stigmas in 5 ml EtOH). Then, centrifuged 10 mins at 13000 g at + 4°C. Supernatants were filtered (0.45 µm PES filter) and divided in 4 tubes, then evaporated to dryness using Speed Vac System for 2h (Concentrator, Eppendorf). Dried extract was kept at -20°C. A stock solution of Saffron Extract (SE) was prepared by dissolving the dried extract in DMSO. The stock solution was diluted using cell culture medium to obtain desired concentration (200 µg/ml- 25 µg/ml).

### 2.2. Characterization of saffron

To determine the quality of our saffron, spectrophotometric analysis was performed as previously described [13,14]. UV-visible spectrophotometry of saffron extracts was acquired as previously described by D’Archivio & Maggi, 2017 [13] using Cary UV-Vis spectrophotometers by Agilent, USA.

### 2.3. Neural senescence *in vitro* model

As cell culture model, human neuroblastoma SH-SY5Y cells were used and cultured as manufacture’s protocol using DMEM supplemented with 10% Fetal bovine serum and 1% Glutamine (ATCC, USA) and maintained at 37 °C with 95% humidified air and 5% CO_2_ (Eppendorf, UK). D-galactose (D-Gal) is a well-known agent to induce the aging process. Based on previous literature [4,15], cells were seeded in a 96 wells plate 7000 cells/well overnight, pretreated with SE (25–200 µg/ml, 24 h) and then exposed to D-Gal (SigmaAldrich, USA; 200 mM, 24 h). Ethical concerns: Although this study did not involve human or animal models, we adhered to ethical guidelines for *in vitro* studies. All experimental procedures were conducted in accordance with the ethical standards and guidelines set forth by the relevant institutional and national research committees.

### 2.4. MTS assay

The MTS colorimetric assay was used for the determination of cell viability in our senescence model. Briefly, cells were plated in a 96-well plate and treated as described above. Then, Cell Titer was added according to manufacturer’s protocols (Promega Corporation Madison, USA). The index of viability, which is dependent on formazan produced, was evaluated using a microplate reader, Infinite F200 (Tecan, Swiss) reading at 492 nm. The assay was performed in quadruplicate.

### 2.5. Live cell imaging cytotox assay

Cytotox Incucyte assay was used for calculating cytotoxic index in live-cell imaging. Cells were plated in a black 96 wells plate and treated as described above. In each condition, 250 nM of IncuCyte Cytotox Red Reagent (Essen BioScience, Newark, UK) were added for counting dead cells. The plate was put in IncuCyte device (20 ×  objectives). The cytotoxicity was recorded (3 images for well) every 3 hours by both phase contrast and fluorescence scanning for 72 h at 37 °C and 5% CO_2_. Images were analyzed utilizing the Incucyte ZOOM software (Newark, UK), and the data were reported as red object count (per image).

### 2.6. β-galactosidase (β-GAL) assay

Senescence-associated β-GAL level is routinely measured as a biomarker for evaluating replicative/induced senescence in cells. β-gal colorimetric assay was performed according to manufacturer’s protocol. Briefly, media was removed, and Pierce β-GAL reagent (ThermoScientific, USA) was added. The plate was incubated for 30 mins at 37° C and β-GAL content was measured at 405 nm using a microplate reader.

### 2.7. ROS assay

To measure ROS production in the *in vitro* model, we used the DCFDA assay (Abcam, UK) following the manufacturer’s instructions. Cells were prepared and treated as described, then incubated with 10 µ M DCFDA for 30 minutes at 37 °C in the dark. After washing with PBS, ROS production was measured by detecting the fluorescent DCF using a Tecan Spark (Tecan, Switzerland) at 485 nm excitation and 535 nm emission. Hydrogen peroxide was used as positive control.

### 2.8. Malondialdehyde assay

The Lipid Peroxidation (MDA) assay (ab118970) offers a convenient method for the sensitive detection of malondialdehyde (MDA). In this assay, MDA in the sample reacts with thiobarbituric acid (TBA) to form an MDA-TBA adduct, which can be quantified fluorometrically with excitation/emission at 532/553 nm. The protocol involves adding TBA solution to the samples and standards, followed by incubation at 95°C for 60 minutes. After cooling the samples in an ice bath for 10 minutes, they are transferred to microplate wells and analyzed using a microplate reader (Spark, Tecan).

### 2.9. SOD enzyme activity

The total SOD activity was measured by an enzymatic method using SOD assay kit purchased from Abcam, UK. SOD activity was assessed by measuring the rate of reduction of WST-1 (2-[4-lodophenyl]-3-[4-nitrophenyl]-5-[2,4-dis-ulfophenyl]-2H-tetrazolium, monosodium salt), which produces a water-soluble formazan dye upon reduction with a superoxide anion. The absorbance of WST-1 was measured at 450 nm. The total measured SOD activity was calculated using the decrease in color development at 450 nm compared to the control.

### 2.10. CAT enzyme activity

CAT enzyme activity was assessed using a specific Kit purchased by Abcam. Specifically, the catalase present in the sample reacts with hydrogen peroxide to produce water and oxygen. The unconverted hydrogen peroxide reacts with a probe to produce a product that can be measured colorimetrically at OD 570 nm. Briefly, samples and standards were prepared and loaded to wells. Hydrogen peroxide was then added into wells and incubated at 25°C for 30 minutes. Then, the stop solution was added followed by the developer mix and incubated 10 minutes at 25°C. Plate was read at OD 570 nm in Spark microplate reader.

### 2.11. Western blotting

Control and treated cells were collected and lysed in ice-cold RIPA buffer (Sigma) with freshly added protease and phosphatase inhibitor cocktails (Thermo Fisher Scientific, Waltham, MA, USA). Protein amount was quantified using BCA assay (Thermo Fisher Scientific). Protein lysates were diluted in sample buffer and a denaturing agent as described by the manufacturer (Thermo Fisher Scientific) and then heated at 70 °C for 10 min in Thermo Block (Eppendorf, Hamburg, Germany). 30 μg of proteins was loaded in gradient precast gel (Invitrogen, USA) and electroblotted onto a polyvinyl difluoride membrane (PVDF, Millipore, Darmstadt, Germany) using a Biorad system. Non-specific binding sites were blocked using 5% milk in TBS-T for 1h at RT. Membranes were then incubated overnight at 4 °C with the following primary antibodies, diluted in blocking buffer: anti-P21 1:1000; anti-CyclinD1 1:200; anti-survivin 1:1000; anti-phospo AKT 1:1000; anti-Actin 1:2000. As secondary antibodies, peroxidase-conjugated anti-rabbit or anti-mouse IgG were used. Immunoreactive bands were visualized by chemiluminescent substrate (Thermo, USA), according to the manufacturer’s protocols, and visualized at Uvtec digital system (Cambridge, UK). The relative densities of the immunoreactive bands were determined and normalized to actin. Values were given as relative units.

### 2.12. RT-PCR

Control and treated cells were collected and lysed in Trizol reagent (Invitrogen) and total RNA was extracted according to the manufacturer’s instructions. RNA concentrations were assessed by Qubit Fluorometers (Thermo Fisher Scientific).

First-strand cDNA synthesis was performed from 1 µg of total RNA using SuperScript™ IV VILO™ Master Mix (Invitrogen) according to the manufacturer’s protocol, the ezDNase enzyme was used to remove any genomic DNA contamination. qRT-PCR was performed in a final volume of 20 µ L including 25 ng of the cDNA product, specific forward and reverse primers (500 nM) and PowerUp SYBR Green Master Mix (Applied Biosystems) according to the supplier’s instructions.

The amplification reaction was performed in an Applied Biosystems 7300 system (ThermoFisher Scientific, Rockford, IL, USA), as follows: 2 min at 50 °C and 2 min at 95 °C, then 40 cycles of 15 s at 95 °C and 1 min at 60 °C. Relative gene expression was calculated by using the 2^DDCt method (Livak et al., 2001); Gapdh was chosen as the reference gene and the Control was used as the calibrator. Two biological replicates were performed, and all samples were carried out in duplicate.

The genes analyzed were: Catalase, Superoxide dismutase 1 (SOD1), Survivin, CHOP, GRP 78.

The primers were provided a) from Bio-Rad (Italy) i.e. Catalase (Unique Assay ID qHsaCED0043914) and SOD1 (Unique Assay ID qHsaCID0008628) b) from Eurofins Genomics (Italy) i.e. CHOP (forward AGG CAC TGA GCG TAT CAT GTT reverse CTG TTT CCG TTT CCT GGT TC), GRP 78 (forward GTT CTT GCC GTT CAA GGT GG reverse TGG TAC AGT AAC AAC TGC ATG), survivin (forward AGA ACT GGC CCT TCT TGG AGG reverse CTT TTT ATG TTC CTC TAT GGG GTC), Gapdh (forward TGC ACC ACC AAC TGC TTA GC reverse GGC ATG GAC TGT GGT CAT GAG).

### 2.13. Label-free holotomography

Cells were seeded in a proper glass-bottom black 96-well plate coated with lysin and treated as described above. Live cell time-lapse images were captured under the same conditions, with a frequency of image acquisition of 10 mins. Cells were inspected using a 3D Cell Explorer-fluo microscope (Nanolive) equipped with a dry 60 × /0.8 objective and CMOS camera Sony. Acquired quantitative holotomografic images were segmented and analyzed with built-in Eve and Steve software (Nanolive). Fiji software was used for the final processing (i.e., cropping, video, *etc*.) of the images.

### 2.14. Statistical analysis

To perform statistical analyses GraphPad Prism (GraphPad Software, Inc., La Jolla, CA, USA) was used. One-way ANOVA with Tukey’s post-hoc multiple comparison test was performed to identify significant differences between groups. *P* values <  0.05 were considered statistically significant. Data are available in a public repository (https://zenodo.org/) https://doi.org/10.5281/zenodo.14918565.

## 3. Results

### 3.1. Saffron extraction and characterization

**Fig 1:**
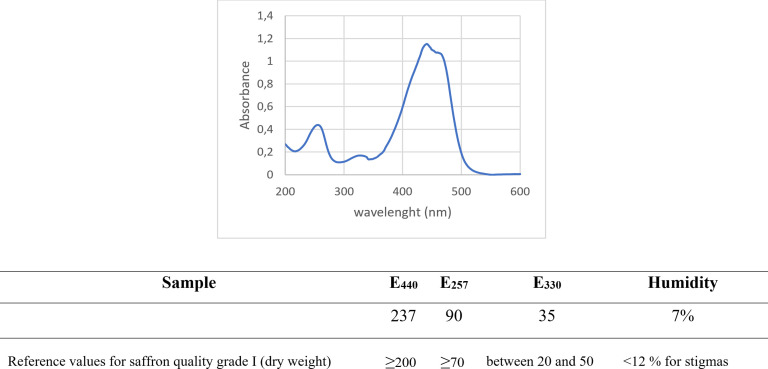
Spectrophotometric analysis UV-Vis.

In the first part of the study, we focused on the extraction and characterization of Saffron Extract derived from Abruzzo region in a hydroalcoholic solution. We then performed a UV-Vis spectrophotometric analysis to determine the quality of our saffron extract. The UV-Vis spectra of saffron extracts (**Fig 1**) showed a strong band that is centered at around 440 nm which derives from the absorption of the polyene conjugated system of crocetin and crocins. The intensities of the secondary bands at 257 nm and 330 nm are ascribed by ISO-3632 Technical Specifications to the contents of picrocrocin and safranal, respectively [16,17]. Nonetheless, the UV spectra also include the absorptions of picrocrocin derivatives (peaking around 250 nm) and flavonoids, primarily kaempferol glycosides (within the range of 265–349 nm) [18]. Crocetin esters exhibit secondary absorption peaks between 250–260 nm for both cis- and trans-isomers, and between 324–327 nm exclusively for cis-crocins [14,18]. As you can appreciate in the table (**Fig 1**), based on ISO-3632.1/2 standard, our saffron stigma are Grade I, highest quality grade of saffron.

### 3.2. Effects on cell viability

**Fig 2:**
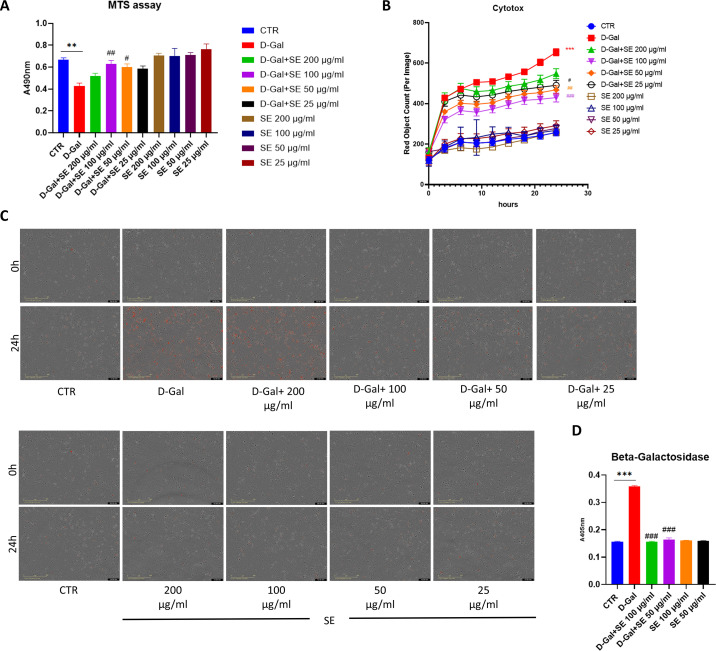
A) MTS assay in human SH-SY5Y upon different treatments at 24h. ANOVA one-way: **, **P** ≤  0.01 vs CTR; ##, **P** ≤  0.01, #, **P** ≤  0.05 vs D-Gal. N = 6; B) Cytotox Incucyte Assay graph 0-24h and **C**) representative images at 0h and 24h in human SH-SY5Y upon different treatments. ANOVA two-way: ***, **P** ≤  0.001 vs CTR; ##, **P** ≤  0.001, ##, **P** ≤  0.01, #, **P** ≤  0.05 vs D-Gal. N = 9. D) Senescence-associated β-GAL levels in human SH-SY5Y upon different treatments; ANOVA two-way: ***, **P** ≤  0.001 vs CTR; ###, **P** ≤  0.001, vs D-Gal. N = 4.

We then tested the hydroalcoholic saffron extract (SE) on an *in vitro* brain senescence model using human SH-SY5Y exposed to D-Gal. Exposure of SH-SY5Y cells to 200mM of D-Gal for 24 hours significantly decreased cell viability. Pretreatment of cells with SE 100 µ g/ml and 50 µ g/ml (24h before exposure to D-Gal) significantly attenuated the effects of D-Gal (p = 0.0078 and p = 0.0322, respectively), as compared to D-Gal-treated cells (**Fig 2A**). Also, treatment of cells with different SE concentrations (25–200 μg/ml) alone for 24 h did not affect cell viability. The concentration range was selected based on previously published literature[4].

In line with these results, exposure of SH-SY5Y cells to 200mM of D-Gal for 24 significantly induced cytotoxicity (***) evaluated by Incucyte live-cell Cytotox assay. As shown in Fig 2B**–**C, pretreatment of cells with SE (at all the tested concentrations), 24h before exposure to D-Gal, significantly attenuated the effects of D-Gal, as compared to D-Gal-treated cells. Also, treatment of cells with different SE concentrations (25–200 μg/ml) alone for 24 h did not show any toxic effect. Thus, for the following experiments we focused on 50 and 100 μg/ml as SE concentration.

### 3.3. Effects on senescence

Senescence-associated β-GAL level is routinely measured as a biomarker for assessing replicative/induced senescence in cells. SH-SY5Y cells treated with D-Gal 200mM showed a significant increase in β-GAL when compared to the control group. Pretreatment of cells with SE at both tested concentrations induced a significant reduction in the senescence marker. SE alone did not affect the senescence marker (**Fig 2D**).

### 3.4. Oxidative stress

**Fig 3:**
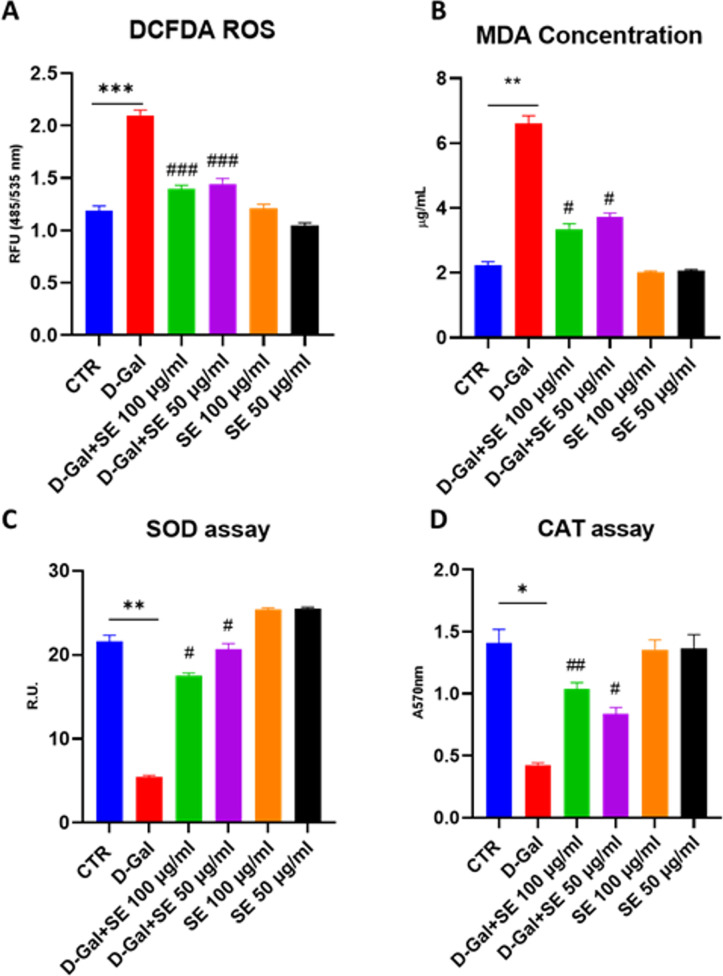
Oxidative stress analysis on D-Gal-induced senescence upon SE treatment. A) ROS assay ANOVA one-way: ***, **P** ≤  0.001 vs CTR; ###, **P** ≤  0.001 vs D-Gal. N = 4; B) MDA levels C) SOD enzymatic activity assay, ANOVA one-way: **, **P** ≤  0.01 vs CTR; ##, **P** ≤  0.01, #, **P** ≤  0.05 vs D-Gal. N = 4. D) CAT enzymatic activity assay, ANOVA one-way: **, **P** ≤  0.01 vs CTR; ##, **P** ≤  0.01, #, **P** ≤  0.05 vs D-Gal. N = 4.

Since oxidative stress is a cause of aging and several studies have shown that D-galactose-induced brain aging is due mainly to increasing oxidative stress. Specifically, we focused on the production of ROS, Superoxide dismutase and Catalase enzyme activities (**Fig 3**), enzymes essential for maintaining cellular redox balance and preventing oxidative damage to DNA, proteins, and lipids. We analyzed the ROS production by DCFDA assay (**Fig 3A**). D-Gal (200mM) notably enhanced ROS level, while preincubation with both tested concentrations of SE remarkably diminished ROS level compared to D-Gal-induced aging (p <  0.001). As reported in **Fig 3B**, D-Gal significantly raised malondialdehyde level, a radical oxidative marker, whereas preincubation with SE (at both tested concentrations) markedly alleviated malondialdehyde levels comparing D-GAL group, thus confirming that pretreatment with SE significantly attenuated oxidative stress, a feature of D-GAL-induced aging. Regarding all the performed assays, SE alone did not affect any parameters.

Interestingly, D-Gal induced a decrease in SOD enzyme activity but also in CAT enzyme activity (Fig 3C**–**D), while SE was able to counteract this effect especially at the highest concentration tested (##), suggesting that SE exerted an antioxidant activity restoring the imbalance typical of senescence. Regarding the expression of SODI, and CAT assayed by RT-PCR both decreased in the senescence model while pretreatment with SE restored control conditions (S1 Fig ).

### 3.5. Effects on cell morphology

**Fig 4:**
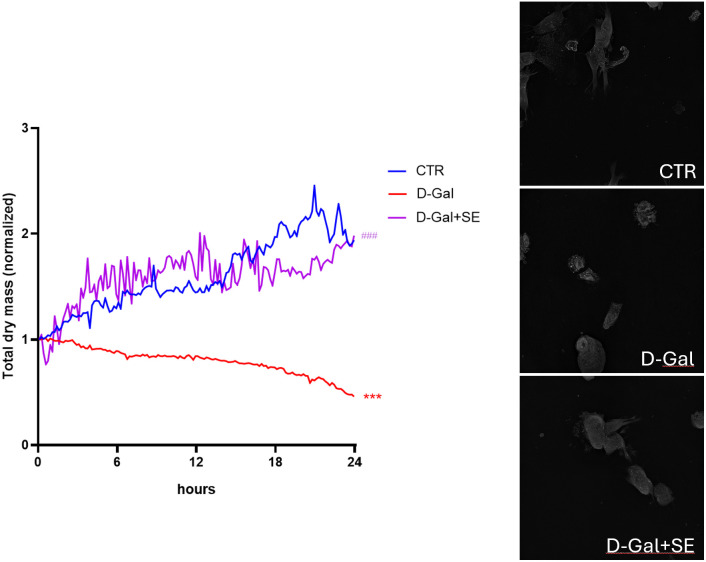
Live-cell label-free holotomographic microscopy. Graph reports Total dry mass of cells upon different conditions. ANOVA one-way: ***, **P** ≤  0.001 vs CTR; ###, **P** ≤  0.001 vs D-Gal. N = 4.

To investigate changes in morphology induced by D-gal in SH-SY5Y cells and the effects of SE, label-free holotomographic microscopy was performed (**Fig 4**). In particular, dry mass, a specific parameter of holotomographic microscopy was evaluated, referring to the dry mass of the cell, therefore all cellular content excluding water (macromolecules), which is very useful for understanding the state of the population under analysis (for example upon stress situations). Treatment with D-gal significantly reduced the total dry mass compared to the CTR group. Meanwhile, pretreatment with SE significantly rescued this parameter compared to the untreated senescent group (**Fig 4**). Moreover, it is possible to clearly observe in the video reported in S1**–**S3 Videos how senescent cells (D-Gal) are less interacting with other cells and showed morphology changes and vacuoles accumulation. Interestingly, the presence of SE was able to ameliorate this condition (D-Gal+SE).

### 3.6. Effects on cell cycle

**Fig 5:**
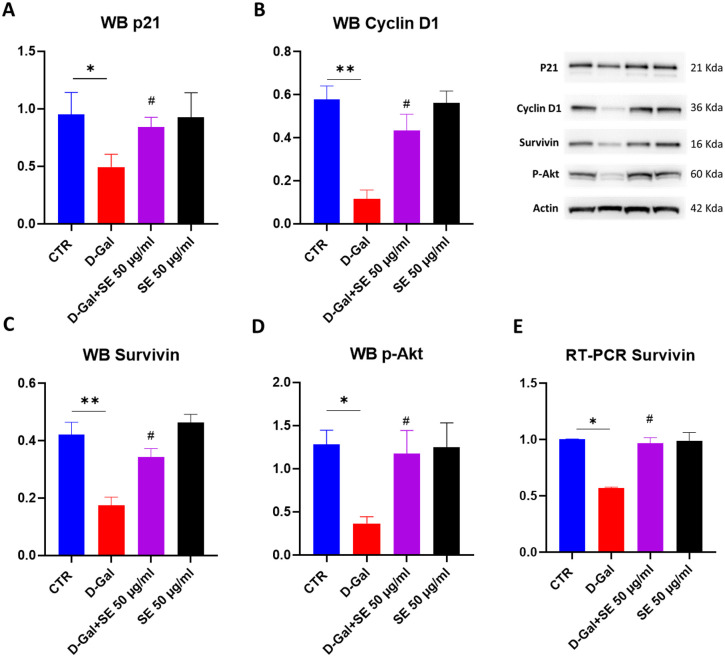
Cell cycle pathway on D-Gal-induced senescence upon SE treatment. **A**) p21 protein levels and representative image. B) Cyclin D1 protein levels and representative image. C) Survivin protein level and representative image. **D**) p-Akt protein levels and representative image. E) RT-PCR of Survivin. ANOVA one-way: ***, **P** ≤  0.001 vs CTR; ###, **P** ≤  0.001 vs D-Gal. N = 3.

We then evaluated by Western blotting the protein levels of P21, one of the most relevant regarding senescence. P21 induction triggers cell cycle arrest in senescent cells, but its activation in senescent cells is only transient; indeed, p21 decreases after the establishment of growth arrest [19,20]. In line with this, in our experimental conditions, p21 protein levels were decreased in senescent cells, while the presence of SE was able to counteract this effect (**Fig 5A**). We also observed a decrease in Cyclin D1 protein level upon D-Gal while SE restored control conditions (**Fig 5B**). Moreover, SE was able to significantly counteract the reduction in Survivin (both in protein levels and expression, **Fig 5C** and E) and the active form of Akt (p-Akt) observed in senescent cells (D-Gal) (**Fig 5D**), suggesting a protective role of SE against D-Gal induced senescence.

### 3.7. Effects on endoplasmic reticulum stress

**Fig 6:**
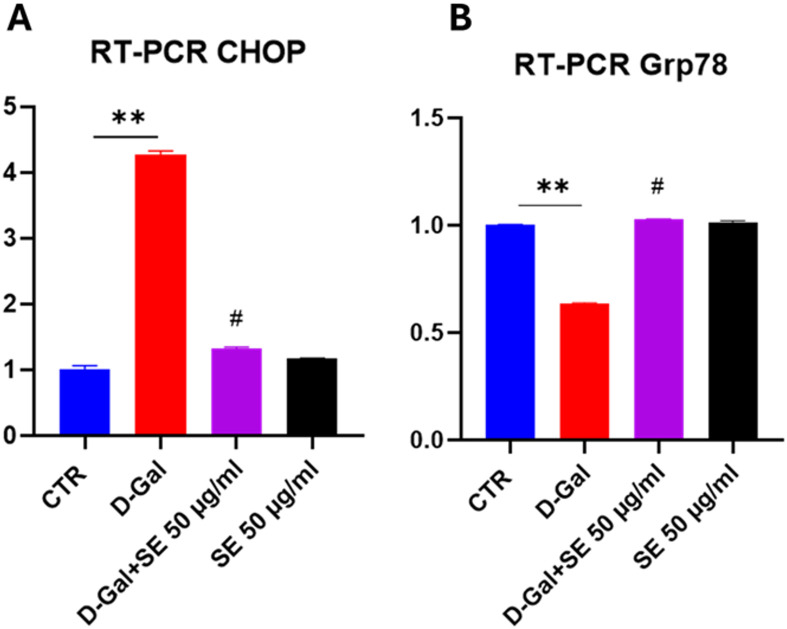
RT-PCR of CHOP and Grp78 on D-Gal-induced senescence upon SE treatment. ANOVA one-way: **, **P** ≤  0.005 vs CTR; #, **P** ≤  0.05 vs D-Gal. N = 3.

Recently, it has been indicated Endoplasmic Reticulum (ER) stress as a driver of brain aging and age-related disorders [21]. Under chronic ER stress, the UPR triggers apoptosis through different mechanisms that concern the upregulation of CHOP, other than the induction of oxidative stress. Glucose Regulated Protein 78 (GRP78) is another key protein in ER stress signaling, whose expression decreases with age [22,23]. Indeed, in our experimental conditions, a significant increase in the expression of CHOP was observed in D-GAL induced senescence, while the pretreatment with SE showed a behavior similar to CTR condition (**Fig 6A**). In parallel, D-GAL induced a decrease in GRP78, and, interestingly, SE was able to reduce its expression (**Fig 6B**). These results suggest that pretreatment with SE counteracted ER stress and promoted cell survival.

## 4. Discussion and conclusions

In the present study, pretreatment with SE exerted an anti-aging effect by regulating the lysosomal enzyme SA-β-gal, reducing neuronal loss, counteracting oxidative and ER stress, in a D-Gal-induced brain aging model. This aligns with recent findings that elevated SA-β-gal activity is a hallmark of brain aging, associated with mitochondrial dysfunction and increased ROS production [24].

Brain senescence is the process of gradual deterioration of brain function and structure due to aging. It is associated with various neurodegenerative diseases, such as Alzheimer’s disease, Parkinson’s disease, and dementia [25]. Brain senescence involves multiple molecular and cellular pathways. Hallmarks of senescence include elevated SA-β-gal activity, mitochondrial dysfunction and higher ROS production, apoptosis and ER stress [2].Saffron is a spice derived from the flower of *Crocus sativus*, which has been used in traditional medicine for various purposes, such as treating depression, anxiety, inflammation, and cancer. Saffron contains several bioactive compounds, such as crocin, crocetin, safranal, and picrocrocin, which have shown neuroprotective effects in preclinical and clinical studies [10]. Recent research demonstrated that saffron extract can counteract cognitive impairment and reduce biomarkers of Alzheimer’s disease by targeting oxidative stress [26]. This supports our findings on the protective effects of saffron extract against oxidative and ER stress.

We used a model of D-gal-induced senescence in SH-SY5Y cells, a widely used model to study the molecular mechanisms of neurodegeneration and aging, and to screen for potential therapeutic agents that can modulate senescence and protect neuronal function [4,6,15]. In our experimental conditions, we observed that senescent cells exhibit several distinctive features including elevated activity of the lysosomal enzyme SA-β-gal, a marker of senescence, as its activity increases in senescent cells due to the accumulation of lysosomes and the shift from oxidative phosphorylation to glycolysis. Our senescent model also showed higher oxidative stress (higher reactive oxygen species production, malondialdehyde parallel with decreased SOD activity and CAT activity), and ER stress (decreased GRP78 and increased CHOP expressions). Pretreatment with SE was found to recover oxidative stress condition and ER stress. Moreover, in the present study, the molecular analysis of aged SH-SY5Y cells showed that SE was able to regulate proteins involved in cell death/survival, including P21, surviving, Akt and cyclin D1.

Overall, our findings suggested that pretreatment with SE was protective against D-GAL-induced senescence, counteracting oxidative and ER stress and proteins that regulate cell death. In parallel, SE was able to ameliorate overall condition and morphology. Nevertheless, brain aging is influenced by other factors as well. These factors may involve inflammatory conditions, protein misfolding, and accumulation. Thus, it will be essential to examine the inflammatory changes in this model in future research.

Additionally, it is important to consider whether saffron crosses the Blood-brain barrier and at what concentration. According to some studies, saffron or its active metabolites, such as crocetin, can cross the BBB and reach the brain, where they may exert neuroprotective effects [9,27]. However, the exact mechanisms and pharmacokinetics of saffron crossing the BBB are still unclear and need further investigation.

This study highlights the potential of saffron extract collected in a specific region of Italy, Abruzzo (central Italy). Future studies should compare this extract with those collected from other regions and countries to evaluate its potential in brain senescence. Finally, these findings suggest that hydroalcoholic saffron extract has potential therapeutic benefits for preventing or delaying neural senescence and its associated disorders. However, additional studies are needed to confirm its safety and efficacy *in vivo* and in humans.

## Supporting information

S1 Fig RT-PCR for CAT and SODI of control cells, cells upon D-Gal and pretreated with saffron and then D-Gal. (PDF)

S1 Video Holotomographic video of control cells (SH-SY5Y). (MP4)

S2 Video Holotomographic video of cells upon D-Gal. (MP4)

S3 Video Holotomographic video of cells pretreated with saffron extract and then D-Gal. (MP4)

## Abbreviations

D-Gal: D-galactose
β-GAL: beta-galactosidase
SE: saffron extract
ROS: reactive oxygen species
AGEs: advanced glycation end products
MDA: malondialdehyde
SOD1: superoxide dismutase 1
