# Supplementary figures and images for: Mechanistic insights into the anti-aging effects of Crocus sativus in a D-Gal-induced in vitro neural senescence model
Source: PLoS One. 2025 Jul 16;20(7):e0320572. doi: 10.1371/journal.pone.0320572 (PMC12266386; doi:10.1371/journal.pone.0320572)

P21

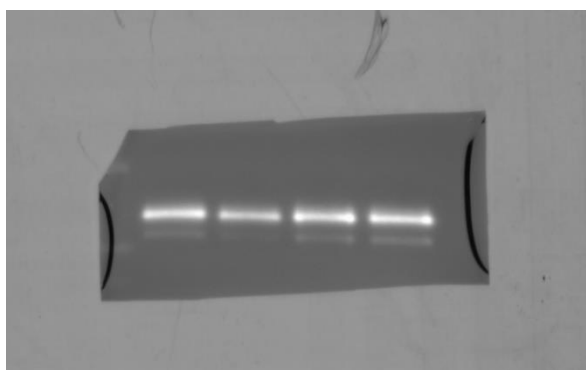

Cyclin D1

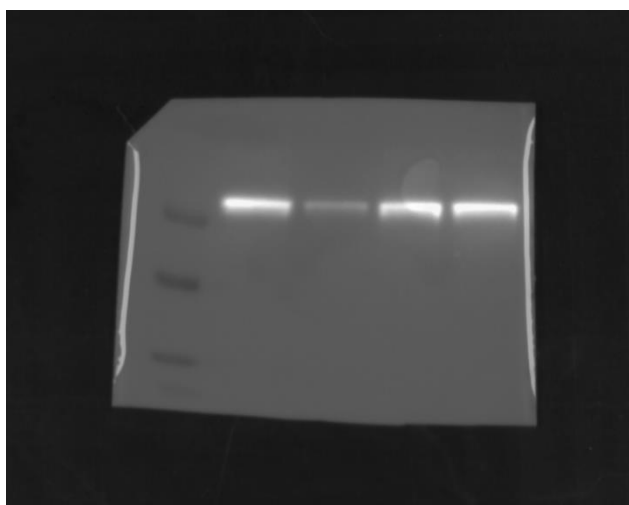

Survivin

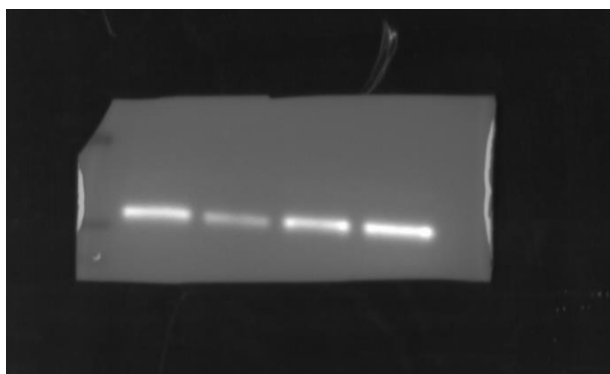

p-AKT

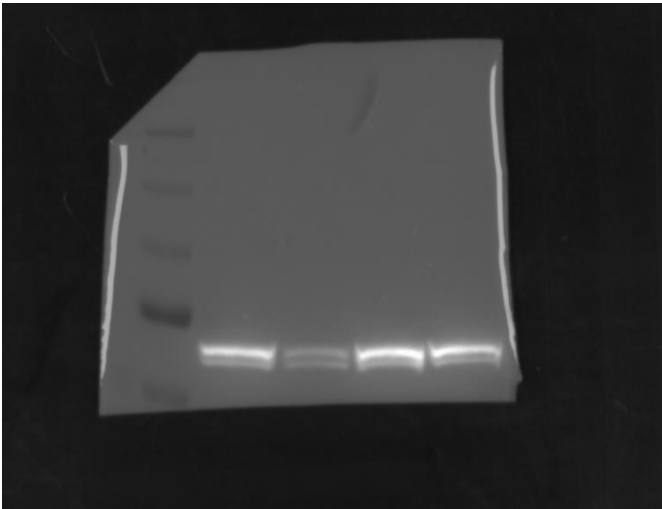

Act

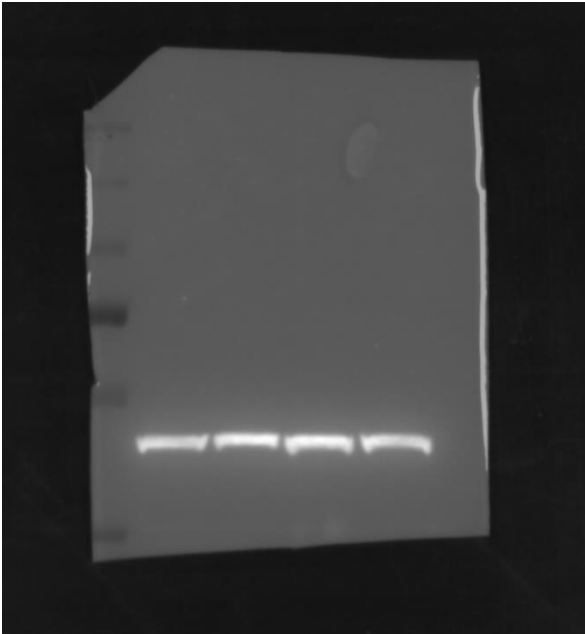

Supplement: S1 Fig — (PDF) [file pone.0320572.s001.pdf]
