# Supplementary material for: Mechanistic insights into the anti-aging effects of Crocus sativus in a D-Gal-induced in vitro neural senescence model
Source: PLoS One. 2025 Jul 16;20(7):e0320572. doi: 10.1371/journal.pone.0320572 (PMC12266386; doi:10.1371/journal.pone.0320572)
Supplement: Attachment — Submitted filename: Response to Reviewers 2.25.docx [file pone.0320572.s006.docx]

Dear Editor,

I am pleased to receive your editorial decision that our manuscript PONE-D-24-50994R1 has been provisionally accepted for publication, pending satisfactory completion of relatively minor points. Please find below my point-by-point responses to the comments, which have been reflected in the revised manuscript (with tracked changes).

**Editorial Feedback:**

1. **Title**: The title is descriptive but could be further optimized for clarity and impact. Consider rephrasing it to: *"Mechanistic Insights into the Anti-Aging Effects of Crocus sativus in a D-GAL-Induced In Vitro Neural Senescence Model"* to emphasize the mechanistic focus.

**Response: We totally agree with the Editor, and we modified the title accordingly.**

2. **Abstract**:
   - The abstract is concise, but some sentences could be more streamlined. Avoid redundancy, such as repeating information about oxidative stress and ER stress in different contexts.
   - Future directions (e.g., testing in animal models) could be emphasized earlier to align with the journal's broader readership.

**Response: We appreciate the comments and modified the Abstract section following the Editor’s advice.**

3. **Figures and Supplementary Materials**:
   - Ensure high-quality resolution for figures; particularly, Figures 1-6 and supplementary videos/images should be clear when enlarged.
   - Mention if the graphical abstract and supplementary materials comply with PLOS ONE’s submission standards.
   - Please include details on the herbarium where the *Crocus sativus* sample has been registered. Specifically, provide the name of the herbarium center and the herbarium accession number for the saffron sample used in the study. This information is critical for reproducibility and traceability.

**Response: We checked the Figures and Video for high quality resolution and replaced it when possible. We added mention about PLOS ONE’s submission standards for graphical abstract and supplementary material and included details about Crocus sativus sample used in this study.**

4. **Language and Style**:
   - Some sentences in the main text, particularly in the methods and discussion, are verbose. Editorial-level language refinement is needed to enhance flow and readability.
   - Acronyms (e.g., ROS, MDA, etc.) are well-defined but should be minimized in non-technical sections for broader comprehension.

**Response: We appreciate the Editor’s comments and have thoroughly checked the manuscript for English language using Ginger and Grammarly software. Regarding acronyms, we have minimized their use in non-technical sections as suggested.**

5. **Ethics Statement**:
   - Although the study did not involve human or animal models, a statement clarifying adherence to ethical guidelines for in vitro studies is recommended.

**Response: we totally agree and added a statement in the Ethical concerns section.**

**Reviewers' comments:**

**Reviewer #1**: Castelli1 and coworkers presented an interesting study: Crocus sativus reveals promising anti-aging effects in D-GAL-induced in vitro model: a mechanistic study

The authors addressed an interesting topic with appropriate methodology, highlighted the strengths of their study, and presented some interesting results and conclusions. Overall, this is a clear, concise, and well-written manuscript. This article covers lots of information. On the one hand, many general statements are made without proper references. Please try to remove some of the general statements or speculations but add some details for some key refs for better illustration.
The authors better use more updated and appropriate references whenever possible.
The introduction is relevant, and theory based. Sufficient information about the previous study findings should be presented for readers to follow the present study rationale and procedures.I suggest that authors would provide some information about uses and documented activities of those molecules, including eventual toxicities
**Response: We thank Reviewer 1 for the time spent revising our research article and for the comments provided, which have greatly improved our manuscript. We have addressed all the points raised and have now included information about the positive and negative effects of saffron molecules as suggested.**

- The methods are generally appropriate,
Can the authors explain the rationale for using the specific concentration range for treatment with Saffron? How do these concentrations compare with physiologically relevant doses in the clinical setting?
Cytotox Icucyte assay was used for calculating cytotoxic index. Could complementary assays, such as real-time impedance monitoring or live dead staining, substantiate the claims of cytotoxicity?

**Response: thank you for the comments. The range of concentrations used was chosen based on previously published papers, and we have now added this information. We completely agree with the reviewer; we selected Cytotox live-cell imaging because it is an unbiased assay and more sensitive compared to typical live/dead staining. Regarding real-time impedance monitoring, it would indeed be interesting, but unfortunately, our lab does not have the instrument to perform it.**

- the results are clear and compared with previous similar studies, the current study has innovations and advantages
For clarity, strengths and limitations should follow the conclusion section; authors should rectify it. The quality of the figures is poor and should be rectified.
The performed study and results could have implication for research and clinical practice in the future. Therefore, the present article can be accepted for publication after minor revision.

**Response: We thank the reviewer for the comments and have modified the manuscript following the suggestions. Additionally, the quality of the figures and video has been checked and replaced with higher quality images.**

**Reviewer #2**: The manuscript entitled "Crocus sativus reveals promising anti-aging effects in D-GAL-induced in vitro model: a mechanistic study" presents data about the potential anti-aging effect of saffron extract using assessment of cell proliferation, live-cell cytotoxicity, Beta-Galactosidase (β-GAL), lipid peroxidation, intracellular reactive oxygen species (ROS), advanced glycation end products (AGEs), malondialdehyde (MDA) levels, cell cycle and endoplasmic reticulum stress. In my opinion, this manuscript would be suitable for publication in this journal after some modification:
**Response: We thank Reviewer 2 for the time spent revising our research article and for the comments provided, which have greatly improved our manuscript. We have addressed all the points raised.**


1. There are some spacing errors in the document. There should be a space between a variable and its unit with the following exceptions: between a variable and the degree (°) or percent (%) signs, and between a variable and the abbreviations for molarity (M and mM). Examples on Page 8 (Line 260 and 269): your “200 mM” should be 200mM.

**Response: We apologize for the oversights. We have now thoroughly checked the manuscript for English and typos.**
2. Introduction should also briefly discuss the main outcomes of the test in present study.

**Response: We totally agree with the Reviewer and added more details.**

3. Please provide brand name of UV-visible spectrophotometry used for determination of the quality of your saffron.
**Response: we apologize for the oversight. We now added the brand name in method section (Cary UV-Vis spectrophotometer, Agilent).**

4. Reference number [19] is too old. It is better to update it.
**Response: We totally agree with the reviewer, and we replaced the reference with a newest one as suggested.**

5. Please provide high resolution figures and graphs.
**Response: We checked all the figures and videos and replaced with the highest resolution possible.**

6. The discussion needs to be strengthened at a deeper level, focusing on the significance of the study. In most parts of discussion section the authors only describe the results of the study, they should discuss about obtained results and compare the obtained results with previous studies. Please add some literature (2021-2024) to explain the research findings.

**Response: We appreciate the Reviewer’s comments and modified the discussion section following his/her suggestions.**

**We would like to extend our gratitude to the Editors and Reviewers for their time and insightful comments, which have significantly improved our manuscript. We hope that the revised manuscript is now suitable for publication in PLOS ONE.**
