# Supplementary material for: Mechanistic insights into the anti-aging effects of Crocus sativus in a D-Gal-induced in vitro neural senescence model
Source: PLoS One. 2025 Jul 16;20(7):e0320572. doi: 10.1371/journal.pone.0320572 (PMC12266386; doi:10.1371/journal.pone.0320572)
Supplement: Peer review history

# Peer review: Decision Letter 0

**DOI:** 10.1371/journal.pone.0320572.r001

**Authors:** Zahra Lorigooini

21 Dec 2024

PONE-D-24-50994Crocus sativus reveals promising anti-aging effects in D-GAL-induced in vitro model: a mechanistic studyPLOS ONE

Dear Dr. Castelli,

Thank you for submitting your manuscript to PLOS ONE. After careful consideration, we feel that it has merit but does not fully meet PLOS ONE’s publication criteria as it currently stands. Therefore, we invite you to submit a revised version of the manuscript that addresses the points raised during the review process.

==============================<h3>**Editorial Feedback:** </h3>

1. **Title** : The title is descriptive but could be further optimized for clarity and impact. Consider rephrasing it to: *"Mechanistic Insights into the Anti-Aging Effects of Crocus sativus in a D-GAL-Induced In Vitro Neural Senescence Model"* to emphasize the mechanistic focus.
2. **Abstract** :
  - The abstract is concise, but some sentences could be more streamlined. Avoid redundancy, such as repeating information about oxidative stress and ER stress in different contexts.
  - Future directions (e.g., testing in animal models) could be emphasized earlier to align with the journal's broader readership.
3. **Figures and Supplementary Materials** :
  - Ensure high-quality resolution for figures; particularly, Figures 1-6 and supplementary videos/images should be clear when enlarged.
  - Mention if the graphical abstract and supplementary materials comply with PLOS ONE’s submission standards.
  - Please include details on the herbarium where the *Crocus sativus* sample has been registered. Specifically, provide the name of the herbarium center and the herbarium accession number for the saffron sample used in the study. This information is critical for reproducibility and traceability.
4. **Language and Style** :
  - Some sentences in the main text, particularly in the methods and discussion, are verbose. Editorial-level language refinement is needed to enhance flow and readability.
  - Acronyms (e.g., ROS, MDA, etc.) are well-defined but should be minimized in non-technical sections for broader comprehension.
5. **Ethics Statement** :
  - Although the study did not involve human or animal models, a statement clarifying adherence to ethical guidelines for in vitro studies is recommended.

==============================

Please submit your revised manuscript by Feb 04 2025 11:59PM. If you will need more time than this to complete your revisions, please reply to this message or contact the journal office at plosone@plos.org . When you're ready to submit your revision, log on to https://www.editorialmanager.com/pone/ and select the 'Submissions Needing Revision' folder to locate your manuscript file.

Please include the following items when submitting your revised manuscript:

- A rebuttal letter that responds to each point raised by the academic editor and reviewer(s). You should upload this letter as a separate file labeled 'Response to Reviewers'.
- A marked-up copy of your manuscript that highlights changes made to the original version. You should upload this as a separate file labeled 'Revised Manuscript with Track Changes'.
- An unmarked version of your revised paper without tracked changes. You should upload this as a separate file labeled 'Manuscript'.

If you would like to make changes to your financial disclosure, please include your updated statement in your cover letter. Guidelines for resubmitting your figure files are available below the reviewer comments at the end of this letter.

If applicable, we recommend that you deposit your laboratory protocols in protocols.io to enhance the reproducibility of your results. Protocols.io assigns your protocol its own identifier (DOI) so that it can be cited independently in the future. For instructions see: https://journals.plos.org/plosone/s/submission-guidelines#loc-laboratory-protocols . Additionally, PLOS ONE offers an option for publishing peer-reviewed Lab Protocol articles, which describe protocols hosted on protocols.io. Read more information on sharing protocols at https://plos.org/protocols?utm_medium=editorial-email&utm_source=authorletters&utm_campaign=protocols .

We look forward to receiving your revised manuscript.

Kind regards,

Zahra Lorigooini

Academic Editor

PLOS ONE

Journal Requirements:

When submitting your revision, we need you to address these additional requirements.

1. Please ensure that your manuscript meets PLOS ONE's style requirements, including those for file naming. The PLOS ONE style templates can be found at 

https://journals.plos.org/plosone/s/file?id=wjVg/PLOSOne_formatting_sample_main_body.pdf and 

https://journals.plos.org/plosone/s/file?id=ba62/PLOSOne_formatting_sample_title_authors_affiliations.pdf

2. PLOS ONE now requires that authors provide the original uncropped and unadjusted images underlying all blot or gel results reported in a submission’s figures or Supporting Information files. This policy and the journal’s other requirements for blot/gel reporting and figure preparation are described in detail at https://journals.plos.org/plosone/s/figures#loc-blot-and-gel-reporting-requirements and https://journals.plos.org/plosone/s/figures#loc-preparing-figures-from-image-files. When you submit your revised manuscript, please ensure that your figures adhere fully to these guidelines and provide the original underlying images for all blot or gel data reported in your submission. See the following link for instructions on providing the original image data: https://journals.plos.org/plosone/s/figures#loc-original-images-for-blots-and-gels.   

In your cover letter, please note whether your blot/gel image data are in Supporting Information or posted at a public data repository, provide the repository URL if relevant, and provide specific details as to which raw blot/gel images, if any, are not available. Email us at plosone@plos.org if you have any questions.

3. We noticed you have some minor occurrence of overlapping text with the following previous publication(s), which needs to be addressed:

- https://www.mdpi.com/1420-3049/25/10/2332 (Text overlap)

In your revision ensure you cite all your sources (including your own works), and quote or rephrase any duplicated text outside the methods section. Further consideration is dependent on these concerns being addressed.

4. In the online submission form, you indicated that The data underlying the results presented in the study are available from corresponding Authors upon reasonable requests.

All PLOS journals now require all data underlying the findings described in their manuscript to be freely available to other researchers, either 1. In a public repository, 2. Within the manuscript itself, or 3. Uploaded as supplementary information.

This policy applies to all data except where public deposition would breach compliance with the protocol approved by your research ethics board. If your data cannot be made publicly available for ethical or legal reasons (e.g., public availability would compromise patient privacy), please explain your reasons on resubmission and your exemption request will be escalated for approval. 

5. Please include captions for your Supporting Information files at the end of your manuscript, and update any in-text citations to match accordingly. Please see our Supporting Information guidelines for more information: http://journals.plos.org/plosone/s/supporting-information.

6. Please review your reference list to ensure that it is complete and correct. If you have cited papers that have been retracted, please include the rationale for doing so in the manuscript text, or remove these references and replace them with relevant current references. Any changes to the reference list should be mentioned in the rebuttal letter that accompanies your revised manuscript. If you need to cite a retracted article, indicate the article’s retracted status in the References list and also include a citation and full reference for the retraction notice.

[Note: HTML markup is below. Please do not edit.]

Reviewers' comments:

Reviewer's Responses to Questions

**Comments to the Author**

1. Is the manuscript technically sound, and do the data support the conclusions?

The manuscript must describe a technically sound piece of scientific research with data that supports the conclusions. Experiments must have been conducted rigorously, with appropriate controls, replication, and sample sizes. The conclusions must be drawn appropriately based on the data presented. 

Reviewer #1: Yes

Reviewer #2: Yes

**********

2. Has the statistical analysis been performed appropriately and rigorously? 

Reviewer #1: Yes

Reviewer #2: Yes

**********

3. Have the authors made all data underlying the findings in their manuscript fully available?

The PLOS Data policy requires authors to make all data underlying the findings described in their manuscript fully available without restriction, with rare exception (please refer to the Data Availability Statement in the manuscript PDF file). The data should be provided as part of the manuscript or its supporting information, or deposited to a public repository. For example, in addition to summary statistics, the data points behind means, medians and variance measures should be available. If there are restrictions on publicly sharing data—e.g. participant privacy or use of data from a third party—those must be specified.

Reviewer #1: Yes

Reviewer #2: Yes

**********

4. Is the manuscript presented in an intelligible fashion and written in standard English?

PLOS ONE does not copyedit accepted manuscripts, so the language in submitted articles must be clear, correct, and unambiguous. Any typographical or grammatical errors should be corrected at revision, so please note any specific errors here.

Reviewer #1: Yes

Reviewer #2: Yes

**********

5. Review Comments to the Author

Please use the space provided to explain your answers to the questions above. You may also include additional comments for the author, including concerns about dual publication, research ethics, or publication ethics. (Please upload your review as an attachment if it exceeds 20,000 characters)

Reviewer #1: Castelli1 and coworkers presented an interesting study: Crocus sativus reveals promising anti-aging effects in D-GAL-induced in vitro model: a mechanistic study

The authors addressed an interesting topic with appropriate methodology, highlighted the strengths of their study, and presented some interesting results and conclusions. Overall, this is a clear, concise, and well-written manuscript. This article covers lots of information. On the one hand, many general statements are made without proper references. Please try to remove some of the general statements or speculations but add some details for some key refs for better illustration.

The authors better use more updated and appropriate references whenever possible.

The introduction is relevant, and theory based. Sufficient information about the previous study findings should be presented for readers to follow the present study rationale and procedures.I suggest that authors would provide some information about uses and documented activities of those molecules, including eventual toxicities

-

- The methods are generally appropriate,

Can the authors explain the rationale for using the specific concentration range for treatment with Saffron? How do these concentrations compare with physiologically relevant doses in the clinical setting?

Cytotox Icucyte assay was used for calculating cytotoxic index. Could complementary assays, such as real-time impedance monitoring or live dead staining, substantiate the claims of cytotoxicity?

- the results are clear and compared with previous similar studies, the current study has innovations and advantages

For clarity, strengths and limitations should follow the conclusion section; authors should rectify it.

The quality of the figures is poor and should be rectified.

The performed study and results could have implication for research and clinical practice in the future. Therefore, the present article can be accepted for publication after minor revision.

Reviewer #2: The manuscript entitled "Crocus sativus reveals promising anti-aging effects in D-GAL-induced in vitro model: amechanistic study" presents data about the potential anti-aging effect of saffron extract using assessment of cell proliferation, live-cell cytotoxicity, Beta-Galactosidase (β-GAL), lipid peroxidation, intracellular reactive oxygen species (ROS), advanced glycation end products (AGEs), malondialdehyde (MDA) levels, cell cycle and endoplasmic reticulum stress. In my opinion, this manuscript would be suitable for publication in this journal after some modification:

1. There are some spacing errors in the document. There should be a space between a variable and its unit with the following exceptions: between a variable and the degree (°) or percent (%) signs, and between a variable and the abbreviations for molarity (M and mM). Examples on Page 8 (Line 260 and 269): your “200 mM” should be 200mM.

2. Introduction should also briefly discuss the main outcomes of the test in present study.

3. Please provide brand name of UV-visible spectrophotometry used for determination of the quality of your saffron.

4. Reference number [19] is too old. It is better to update it.

5. Please provide high resolution figures and graphs.

6. The discussion needs to be strengthened at a deeper level, focusing on the significance of the study. In most parts of discussion section the authors only describe the results of the study, they should discuss about obtained results and compare the obtained results with previous studies. Please add some literature (2021-2024) to explain the research findings.

**********

6. PLOS authors have the option to publish the peer review history of their article (what does this mean? ). If published, this will include your full peer review and any attached files.

If you choose “no”, your identity will remain anonymous but your review may still be made public.

**Do you want your identity to be public for this peer review?** For information about this choice, including consent withdrawal, please see our Privacy Policy .

Reviewer #1: No

Reviewer #2: **Yes: ** Mohammad Hadi Zarei

**********

[NOTE: If reviewer comments were submitted as an attachment file, they will be attached to this email and accessible via the submission site. Please log into your account, locate the manuscript record, and check for the action link "View Attachments". If this link does not appear, there are no attachment files.]

While revising your submission, please upload your figure files to the Preflight Analysis and Conversion Engine (PACE) digital diagnostic tool, https://pacev2.apexcovantage.com/ . PACE helps ensure that figures meet PLOS requirements. To use PACE, you must first register as a user. Registration is free. Then, login and navigate to the UPLOAD tab, where you will find detailed instructions on how to use the tool. If you encounter any issues or have any questions when using PACE, please email PLOS at figures@plos.org . Please note that Supporting Information files do not need this step.

# Author comment: Author response to Decision Letter 1

**DOI:** 10.1371/journal.pone.0320572.r002

4 Feb 2025

Dear Editor,

I am pleased to receive your editorial decision that our manuscript PONE-D-24-50994R1 has been provisionally accepted for publication, pending satisfactory completion of relatively minor points. Please find below my point-by-point responses to the comments, which have been reflected in the revised manuscript (with tracked changes).

Editorial Feedback:

1. Title: The title is descriptive but could be further optimized for clarity and impact. Consider rephrasing it to: "Mechanistic Insights into the Anti-Aging Effects of Crocus sativus in a D-GAL-Induced In Vitro Neural Senescence Model" to emphasize the mechanistic focus.

Response: We totally agree with the Editor, and we modified the title accordingly.

2. Abstract:

o The abstract is concise, but some sentences could be more streamlined. Avoid redundancy, such as repeating information about oxidative stress and ER stress in different contexts.

o Future directions (e.g., testing in animal models) could be emphasized earlier to align with the journal's broader readership.

Response: We appreciate the comments and modified the Abstract section following the Editor’s advice.

3. Figures and Supplementary Materials:

o Ensure high-quality resolution for figures; particularly, Figures 1-6 and supplementary videos/images should be clear when enlarged.

o Mention if the graphical abstract and supplementary materials comply with PLOS ONE’s submission standards.

o Please include details on the herbarium where the Crocus sativus sample has been registered. Specifically, provide the name of the herbarium center and the herbarium accession number for the saffron sample used in the study. This information is critical for reproducibility and traceability.

Response: We checked the Figures and Video for high quality resolution and replaced it when possible. We added mention about PLOS ONE’s submission standards for graphical abstract and supplementary material and included details about Crocus sativus sample used in this study.

4. Language and Style:

o Some sentences in the main text, particularly in the methods and discussion, are verbose. Editorial-level language refinement is needed to enhance flow and readability.

o Acronyms (e.g., ROS, MDA, etc.) are well-defined but should be minimized in non-technical sections for broader comprehension.

Response: We appreciate the Editor’s comments and have thoroughly checked the manuscript for English language using Ginger and Grammarly software. Regarding acronyms, we have minimized their use in non-technical sections as suggested.

5. Ethics Statement:

o Although the study did not involve human or animal models, a statement clarifying adherence to ethical guidelines for in vitro studies is recommended.

Response: we totally agree and added a statement in the Ethical concerns section.

Reviewers' comments:

Reviewer #1: Castelli1 and coworkers presented an interesting study: Crocus sativus reveals promising anti-aging effects in D-GAL-induced in vitro model: a mechanistic study

The authors addressed an interesting topic with appropriate methodology, highlighted the strengths of their study, and presented some interesting results and conclusions. Overall, this is a clear, concise, and well-written manuscript. This article covers lots of information. On the one hand, many general statements are made without proper references. Please try to remove some of the general statements or speculations but add some details for some key refs for better illustration.

The authors better use more updated and appropriate references whenever possible.

The introduction is relevant, and theory based. Sufficient information about the previous study findings should be presented for readers to follow the present study rationale and procedures.I suggest that authors would provide some information about uses and documented activities of those molecules, including eventual toxicities

Response: We thank Reviewer 1 for the time spent revising our research article and for the comments provided, which have greatly improved our manuscript. We have addressed all the points raised and have now included information about the positive and negative effects of saffron molecules as suggested.

- The methods are generally appropriate,

Can the authors explain the rationale for using the specific concentration range for treatment with Saffron? How do these concentrations compare with physiologically relevant doses in the clinical setting?

Cytotox Icucyte assay was used for calculating cytotoxic index. Could complementary assays, such as real-time impedance monitoring or live dead staining, substantiate the claims of cytotoxicity?

Response: thank you for the comments. The range of concentrations used was chosen based on previously published papers, and we have now added this information. We completely agree with the reviewer; we selected Cytotox live-cell imaging because it is an unbiased assay and more sensitive compared to typical live/dead staining. Regarding real-time impedance monitoring, it would indeed be interesting, but unfortunately, our lab does not have the instrument to perform it.

- the results are clear and compared with previous similar studies, the current study has innovations and advantages

For clarity, strengths and limitations should follow the conclusion section; authors should rectify it. The quality of the figures is poor and should be rectified.

The performed study and results could have implication for research and clinical practice in the future. Therefore, the present article can be accepted for publication after minor revision.

Response: We thank the reviewer for the comments and have modified the manuscript following the suggestions. Additionally, the quality of the figures and video has been checked and replaced with higher quality images.

Reviewer #2: The manuscript entitled "Crocus sativus reveals promising anti-aging effects in D-GAL-induced in vitro model: a mechanistic study" presents data about the potential anti-aging effect of saffron extract using assessment of cell proliferation, live-cell cytotoxicity, Beta-Galactosidase (β-GAL), lipid peroxidation, intracellular reactive oxygen species (ROS), advanced glycation end products (AGEs), malondialdehyde (MDA) levels, cell cycle and endoplasmic reticulum stress. In my opinion, this manuscript would be suitable for publication in this journal after some modification:

Response: We thank Reviewer 2 for the time spent revising our research article and for the comments provided, which have greatly improved our manuscript. We have addressed all the points raised.

1. There are some spacing errors in the document. There should be a space between a variable and its unit with the following exceptions: between a variable and the degree (°) or percent (%) signs, and between a variable and the abbreviations for molarity (M and mM). Examples on Page 8 (Line 260 and 269): your “200 mM” should be 200mM.

Response: We apologize for the oversights. We have now thoroughly checked the manuscript for English and typos.

2. Introduction should also briefly discuss the main outcomes of the test in present study.

Response: We totally agree with the Reviewer and added more details.

3. Please provide brand name of UV-visible spectrophotometry used for determination of the quality of your saffron.

Response: we apologize for the oversight. We now added the brand name in method section (Cary UV-Vis spectrophotometer, Agilent).

4. Reference number [19] is too old. It is better to update it.

Response: We totally agree with the reviewer, and we replaced the reference with a newest one as suggested.

5. Please provide high resolution figures and graphs.

Response: We checked all the figures and videos and replaced with the highest resolution possible.

6. The discussion needs to be strengthened at a deeper level, focusing on the significance of the study. In most parts of discussion section the authors only describe the results of the study, they should discuss about obtained results and compare the obtained results with previous studies. Please add some literature (2021-2024) to explain the research findings.

Response: We appreciate the Reviewer’s comments and modified the discussion section following his/her suggestions.

We would like to extend our gratitude to the Editors and Reviewers for their time and insightful comments, which have significantly improved our manuscript. We hope that the revised manuscript is now suitable for publication in PLOS ONE.

________________________________________

Attachment Submitted filename: Response to Reviewers 2.25.docx

# Peer review: Decision Letter 1

**DOI:** 10.1371/journal.pone.0320572.r003

**Authors:** Zahra Lorigooini

21 Feb 2025

Mechanistic insights into the anti-aging effects of Crocus sativus in a D-Gal-induced in vitro neural senescence model

PONE-D-24-50994R1

Dear Dr. Castelli,

We’re pleased to inform you that your manuscript has been judged scientifically suitable for publication and will be formally accepted for publication once it meets all outstanding technical requirements.

Within one week, you’ll receive an e-mail detailing the required amendments. When these have been addressed, you’ll receive a formal acceptance letter and your manuscript will be scheduled for publication.

An invoice will be generated when your article is formally accepted. Please note, if your institution has a publishing partnership with PLOS and your article meets the relevant criteria, all or part of your publication costs will be covered. Please make sure your user information is up-to-date by logging into Editorial Manager at Editorial Manager®  and clicking the ‘Update My Information' link at the top of the page. If you have any questions relating to publication charges, please contact our Author Billing department directly at authorbilling@plos.org.

If your institution or institutions have a press office, please notify them about your upcoming paper to help maximize its impact. If they’ll be preparing press materials, please inform our press team as soon as possible -- no later than 48 hours after receiving the formal acceptance. Your manuscript will remain under strict press embargo until 2 pm Eastern Time on the date of publication. For more information, please contact onepress@plos.org.

Kind regards,

Zahra Lorigooini

Academic Editor

PLOS ONE

Additional Editor Comments (optional):

Reviewers' comments:

Reviewer's Responses to Questions

**Comments to the Author**

1. If the authors have adequately addressed your comments raised in a previous round of review and you feel that this manuscript is now acceptable for publication, you may indicate that here to bypass the “Comments to the Author” section, enter your conflict of interest statement in the “Confidential to Editor” section, and submit your "Accept" recommendation.

Reviewer #2: All comments have been addressed

**********

2. Is the manuscript technically sound, and do the data support the conclusions?

The manuscript must describe a technically sound piece of scientific research with data that supports the conclusions. Experiments must have been conducted rigorously, with appropriate controls, replication, and sample sizes. The conclusions must be drawn appropriately based on the data presented. 

Reviewer #2: Yes

**********

3. Has the statistical analysis been performed appropriately and rigorously? 

Reviewer #2: Yes

**********

4. Have the authors made all data underlying the findings in their manuscript fully available?

The PLOS Data policy requires authors to make all data underlying the findings described in their manuscript fully available without restriction, with rare exception (please refer to the Data Availability Statement in the manuscript PDF file). The data should be provided as part of the manuscript or its supporting information, or deposited to a public repository. For example, in addition to summary statistics, the data points behind means, medians and variance measures should be available. If there are restrictions on publicly sharing data—e.g. participant privacy or use of data from a third party—those must be specified.

Reviewer #2: Yes

**********

5. Is the manuscript presented in an intelligible fashion and written in standard English?

PLOS ONE does not copyedit accepted manuscripts, so the language in submitted articles must be clear, correct, and unambiguous. Any typographical or grammatical errors should be corrected at revision, so please note any specific errors here.

Reviewer #2: Yes

**********

6. Review Comments to the Author

Please use the space provided to explain your answers to the questions above. You may also include additional comments for the author, including concerns about dual publication, research ethics, or publication ethics. (Please upload your review as an attachment if it exceeds 20,000 characters)

Reviewer #2: (No Response)

**********

7. PLOS authors have the option to publish the peer review history of their article (what does this mean? ). If published, this will include your full peer review and any attached files.

If you choose “no”, your identity will remain anonymous but your review may still be made public.

**Do you want your identity to be public for this peer review?** For information about this choice, including consent withdrawal, please see our Privacy Policy .

Reviewer #2: **Yes: ** Mohammad Hadi Zarei

**********

# Editor report: Acceptance letter

**DOI:** 10.1371/journal.pone.0320572.r004

**Authors:** Zahra Lorigooini

PONE-D-24-50994R1

PLOS ONE

Dear Dr. Castelli,

I'm pleased to inform you that your manuscript has been deemed suitable for publication in PLOS ONE. Congratulations! Your manuscript is now being handed over to our production team.

At this stage, our production department will prepare your paper for publication. This includes ensuring the following:

* All references, tables, and figures are properly cited

* All relevant supporting information is included in the manuscript submission,

* There are no issues that prevent the paper from being properly typeset

If revisions are needed, the production department will contact you directly to resolve them. If no revisions are needed, you will receive an email when the publication date has been set. At this time, we do not offer pre-publication proofs to authors during production of the accepted work. Please keep in mind that we are working through a large volume of accepted articles, so please give us a few weeks to review your paper and let you know the next and final steps.

Lastly, if your institution or institutions have a press office, please let them know about your upcoming paper now to help maximize its impact. If they'll be preparing press materials, please inform our press team within the next 48 hours. Your manuscript will remain under strict press embargo until 2 pm Eastern Time on the date of publication. For more information, please contact onepress@plos.org.

If we can help with anything else, please email us at customercare@plos.org.

Thank you for submitting your work to PLOS ONE and supporting open access.

Kind regards,

PLOS ONE Editorial Office Staff

on behalf of

Prof. Zahra Lorigooini

Academic Editor

PLOS ONE
